# Mesenteric CD103^+^DCs Initiate Switched Coxsackievirus B3 VP1-Specific IgA Response to Intranasal Chitosan-DNA Vaccine Through Secreting BAFF/IL-6 and Promoting Th17/Tfh Differentiation

**DOI:** 10.3389/fimmu.2018.02986

**Published:** 2018-12-18

**Authors:** Haoxin Zhao, Jie Yang, Qian Qian, Manli Wu, Min Li, Wei Xu

**Affiliations:** Jiangsu Key Laboratory of Infection and Immunity, Institutes of Biology and Medical Sciences, Soochow University, Suzhou, China

**Keywords:** IgA, class switch recombination (CSR), chitosan, CD103^+^DC, TACI

## Abstract

Intranasal chitosan-formulated DNA vaccination promotes IgA secretion in the intestine. However, the mechanism whereby chitosan-DNA skews IgA class switch recombination (CSR) of B cells in the Gut-associated lymph tissue (GALT) is not fully resolved. In this study, we investigated the effects of nasally administered chitosan-DNA (pcDNA3.1-VP1 plasmid encoding VP1 capsid protein of Coxsackievirus B3) on IgA production, DC activation and Tfh/Th17 response in the intestine. Compared to DNA immunization, intranasal chitosan-DNA vaccination induced antigen-specific IgA production in feces, a pronounced switching of antigen-specific IgA^+^ plasmablast B cells in the mesenteric lymph nodes (MLNs) and an enhanced expression of post-recombination Iα-CH transcripts/IgA germline transcript (αGT) as well as activation-induced cytidine deaminase (AID) in MLN B cells. MLN Tfh frequency was markedly enhanced by chitosan-DNA, and was associated with VP1-specific IgA titer. 24 h after immunization, intranasal chitosan-DNA induced a recruitment of CD103^+^DCs into the MLN that paralleled a selective loss of CD103^+^DCs in the lamina propria (LP). *In vivo* activated MLN-derived CD103^+^DCs produced high levels of IL-6 and BAFF in response to chitosan-DNA, which up-regulated transmembrane activator and CAML interactor (TACI) expression on MLN B cells. Upon co-culture with IgM^+^B in the presence of chitosan-DNA, MLN CD103^+^DCs induced IgA production in a T-dependent manner; and this IgA-promoting effect of CD103^+^DC was blocked by targeting TACI and, to a lower extent, by blocking IL-6. MLN CD103^+^DCs displayed an enhanced capacity to induce an enhanced CD4^+^Th17 response *in vivo* and *in vitro*, and IL-17A deficient mice had a pronounced reduction of specific intestinal IgA following immunization. Taken together, mesenteric CD103^+^DCs are indispensable for the adjuvant activity of chitosan in enhancing DNA vaccine-specific IgA switching in gut through activating BAFF-TACI and IL-6-IL-6R signaling, and through inducing Th17/Tfh differentiation in the MLN.

## Introduction

The predominant immunoglobulin at the mucosal surfaces is secretory IgA (SIgA) serving as the first defense against pathogens through immune exclusion. It also plays important roles in establishing a healthy microbiota and maintaining mucosal homeostasis (1, 2). Impaired IgA production is associated with recurrent respiratory and gastrointestinal infections as well as allergy seen in patients with common variable immunodeficiency (CVID) and hyper-IgM (HIGM) syndrome (3). Polymeric immunoglobulin receptor (pIgR) deficient mice which lack SIgA, lose their protection against cholera toxin (CT) after oral immunization with *Salmonella typhimurium* (4). Passive transfer of IgA and IgA depletion assays demonstrate the protective role of mucosal SIgA against influenza virus and Sendai virus infection (5). SIgA could neutralize intracellular pathogens. Mucosal application of anti-human immunodeficiency virus (HIV) envelope dimeric IgA1 provides potent protection against mucosal transmission of HIV-1 (6). An insufficient induction of effector T cells and SIgA in the lung at the time of *Mycobacterium tuberculosis* (M.tb) infection is also suggested as one of the limitations of BCG vaccine (7). In this regard, mucosal immunization with vaccine antigens, or mucosal passive application of pathogen-specific SIgAs at the mucosa where is the initial entry site for most infectious agents, can be effective alternatives to achieve mucosal protection against severe mucus-related infectious disease (8).

IgA generation is potentiated by class switch recombination (CSR) of the B cells (9). CSR is induced by both T cell-dependent (TD) and -independent (TI) pathways. High-affinity IgA emerges from follicular B cells in Peyer's patches (PP) and mesenteric lymph nodes (MLN) after recognizing microbial toxins and pathogens via TD pathways, whereas intestinal commensals stimulate extra-follicular B cells to generate low-affinity IgA via TI pathways (10). In the TD pathway, CD40 signaling from CD4^+^Th cells is critical for generation of germinal centers (GCs) and induction of activation-induced cytidine deaminase (AID), an essential DNA-editing enzyme directing CSR of B cells, in GC B cells (11). DCs are key contributors to induction and regulation of IgA CSR and differentiation of B cells into IgA plasma cells (PCs) in the intestine mucosa. Upon sampling antigen directly or from M cells, DCs migrate to PPs or to the draining MLN to establish cognate interactions with CD4^+^T cells, inducing Th2, regulatory T cell (Treg), and T follicular helper (Tfh) cells that activate follicular B cells and initiate IgA responses via CD40L and cytokines (TGF-β, IL-4, IL-10, and IL-21) (12). Meanwhile, various DC subsets release TGF-β, IL-10, retinoic acid (RA), nitric oxide (NO), activating factor of the TNF family (BAFF) and a proliferation-inducing ligand (APRIL) by TLRs stimulation which activate B cells by binding with receptors on B cells such as BAFF-R, B cell maturation antigen (BCMA) or transmembrane activator and CAML receptor (TACI) (13, 14).

CD103^+^DCs are the most abundant intestinal DC subset (15). Gut CD103^+^DCs comprise two major subsets, CD103^+^CD11b^−^ cDC1s and CD103^+^CD11b^+^ cDC2s. CD103^+^CD11b^+^DCs are the major subsets in the SI-lamina propria (LP) and the major migratory DC subset (16). They present oral antigens and induce the differentiation of T cells into CD4^+^Foxp3^+^Treg via generating TGF-β and RA (17), while CD103^+^CD11b^−^ DCs are the dominant population in the PPs and colon LP. Both human CD103^+^DC subsets induce Th17 polarization (18) while murine CD103^+^CD11b^+^DCs support Th17 differentiation in the MLN by producing IL-6 (19). CD103^+^DCs-mediated enhancement of local Treg differentiation is associated with a pronounced switching of specific B cells to IgA in the MLN (20). Also, antigen-specific Th17 response is associated with enhanced neutralizing mucosal IgA exclusively after mucosal immunization (21). Although studies have assessed the function of intestinal CD103^+^DCs *in vitro*, their roles in intestinal adaptive immune responses *in vivo* and their involvement in the IgA CSR of intestinal B cells to vaccine antigens and adjuvants are not fully clarified.

In our previous work, formulation DNA (pcDNA3.1-VP1 plasmid encoding VP1 capsid protein of Coxsackievirus B3) vaccine with chitosan, a natural cationic polysaccharide deacetylated from chitin, efficiently promotes the protection against CVB3 infection via eliciting considerable enhanced IgA and T response at the intestine (22–24). Chitosan has been widely used as mucosal carrier for vaccines and as ideal adjuvant (25). Regarding its mucosal IgA-enhancing mechanism, some researchers attribute it to the macrophage-modulatory effect of chitosan (26). Recent studies indicate that chitosan engages the cGAS-STING pathway to trigger antigen specific Th1 and IgG2c responses following chitosan-protein vaccination (27). A combination of chitosan with CpG, TLR9 agonist, induces NLRP3-dependent antigen-specific Th1 and Th17 responses (28). Chitosan substrate culture of DCs up-regulates CD80/86 expression on DC and enhances IL-12 secretion by DCs which promotes the induction of anti-tumor IFNγ^+^CTL activity in mice (29). Chitosan-DNA nanoparticles, prepared by coacervation of chitosan and DNA, are known to elevate mucosal SIgA induction (30) by stimulating maturation and function of DC (31, 32). Nevertheless, how chitosan-formulated DNA engages intestinal DC subsets to activate specific intestinal B cells which undergo class switching to SIgA remains largely unexplored.

In the present study, to elucidate cellular and molecular mechanisms underlying the effects of chitosan-DNA on intestinal DCs and antigen-specific IgA production, mice were intranasally (i.n.) immunized with chitosan-DNA and the targeted DC subsets and IgA-producing B response in the intestine were studied. We show that i.n. immunization with chitosan-DNA induces an enhanced recruitment of CD103^+^DCs into the MLNs, which markedly increases the differentiation of GC Tfh cells, Ag-specific IgA^+^ plasmablast and plasma cells. CD4^+^Th17 response is preferentially induced by chitosan-DNA *in vivo* and *in vitro*, which is required for the Ag-specific IgA production in the intestine.

## Materials and Methods

### Mice and Peptide

Six to eight weeks old male BALB/c mice were obtained from Slac Laboratory Animal (Shanghai, China) and housed under pathogen-free conditions at the Soochow University Laboratory Animal Center. IL-17A knockout mice were a gift from Prof Chen Dong (Tsinghua University, China). Animal experiments were performed in accordance with the Institutional Animal Care and Use Committee of Soochow University. All research protocols were approved by the Animal Ethical Committee of Soochow University (SYXK2015-0058). CVB3 VP1_237−249_ Peptide (FKPKHVKAWIPRP) was synthesized by GL Biochem Corp (Shanghai, China) with purity over 95%.

### Intranasal Chitosan-DNA Immunization

Chitosan-DNA(pcDNA3.1-VP1) complex was prepared as described previously (22, 23). Equal volumes of chitosan solution (Fluka BioChemika, 0.02%, w/v in 5 mM NaAc-HAc buffer, pH 5.5) and DNA solution (400 ug/ml in 5 mM Na_2_SO_4_) were heated to 55°C and then vigorously mixed for 30 s. After anesthetization with 0.75% pentobarbital sodium, mice were intranasally immunized with chitosan-DNA (50 μg), DNA, chitosan or PBS. This procedure was conducted on day 0, 14, 28, and 42. Fecal extracts were prepared by mashing feces pellets in PBS containing a protease inhibitor cocktail (5% non-fat milk, 1 ug/ml aprotinin, 1 mM PMSF) at final concentration of 100 mg/ml before centrifuging at 12,000 rpm for 10 min. Supernatants were collected and stored at −80°C for further analysis.

### ELISPOT Analysis

The filtration plate (Millipore) was coated overnight at 4°C with 10 ug/ml VP1_237−249_ peptide. After washing, the plate was blocked with complete RPMI-1640 for 2 h. Splenocytes or GALT lymphocytes were applied (5 × 10^5^/well) and incubated for 6 h at 37°C with LPS (100 ng/ml, Sigma-Aldrich) as positive control. After removing the supernatant, ice-cold deionized water was added and incubated on ice for 10 min to lyse the remaining cells. After washing, alkaline phosphatase-conjugated goat anti-mouse IgA (Southern Biotech) was added and incubated overnight. The enzyme activity was revealed by using Sigma Fast BCIP/NBT (Sigma-Aldrich). Spots were counted using ImmunoSpot analyzer 5.1.36 software (Cellular Technology).

### IgA and Cytokine Measurements

Total IgA levels were assessed using an ELISA kit (Bethyl Laboratory, United States) according to the manufacturer's instructions. VP1-specific IgA levels were evaluated using an ELISA in accordance with previously published methods (33). Instead of the primary antibody in the mouse IgA ELISA kit, VP1_237−249_ peptide was suspended in 0.05 M carbonate-bicarbonate buffer (pH 9.6) and coated the ELISA plate at 10 μg/ml. Subsequently, the kit was used according to the manufacturer's instructions. Mouse IL-17A, IL-6, IL-12p40, IL-12p70, IL-23p19, and IFNγ in the culture supernatants were quantified using ELISA kits (R&D Systems, MN, United States) in accordance with the manufacturer's instructions. Mouse BAFF was quantified by ELISA kit (Abcam, Cambridge, United Kingdom).

### Isolation of Tissue Lymphocytes

Single-cell suspensions of spleen lymphocytes were obtained by gently pressing the spleen against a 70-μm strainer and then removing erythrocytes by brief exposure to 0.16 M NH_4_Cl at room temperature for 3 min. PP and MLN lymphocytes were prepared as previously reported, with some modifications (34). Both PP and MLN cells were digested with collagenase Type IV (0.5 mg/ml, Sigma-Aldrich) and DNase I (5 μg/ml, Sigma-Aldrich) for 30 min and made into single-cell suspensions. For isolation of LP lymphocytes, small intestines were removed after excluding PPs and then cut into 1-cm pieces and opened up. They were minced in Mg^2+^/Ca^2+^-free HBSS supplemented with 2% FBS, and then 2.5 mM EDTA to remove mucus and epithelial cells at 37°C for 30 min. Tissues were then digested with RPMI 1,640–5% FBS with 1 mg/mL collagenase IV and 5 μg/ml DNase I for 45 min at 37°C under gentle shaking. After filtering through a 70-μm mesh the LP lymphocytes were isolated at the 40–70% interface of a discontinuous Percoll (GE Healthcare, UK) gradient and resuspended in RPMI 1640 medium after centrifugation at 800 × g for 20 min.

### Induction of IgA Production by DC-B-Th Cell Co-culture

BALB/c mice were immunized with chitosan-DNA (50 μg, i.n.) for 24 h. MLN single-cell suspension were prepared as described earlier. MLN CD11c^+^MHCII^+^DCs were sorted on the basis of their expression of CD103 using a FACSAria (BD Biosciences) (>95% purity). Naive MLN B cells were sorted on the basis of their expression of IgM and CD19 using a FACSAria. Naive CD4^+^CD62L^high^ T cells from MLN of mice were prepared using MACS beads (Miltenyi Biotec, Germany). For *in vitro* IgA switching, naïve B cells (5 × 10^5^) were co-cultured with naïve CD4^+^Th0 cells (5 × 10^5^) and with MLN CD103^+^DCs (2.5 × 10^4^) in the presence of DNA or chitosan-DNA (5 ug/ml) for 4 days after which VP1_237−249_ peptide was added for another 3 days. Neutralizing Ab against IL-6 (10 μg/ml, BD PharMingen), TACI (5 μg/ml, R&D system) or rat IgG1 isotype control (10 μg/ml, BD PharMingen) was added to cultures. VP1-specific IgA in the culture supernatants was quantified by ELISA.

### Flow Cytometric Analysis

Flow cytometry Abs were purchased from BD Pharmingen, e-Bioscience and Biolegend (San Diego, CA). Single-cell suspensions, after anti-CD16/CD32 blocking, were stained for 25 min at 4°C with the following primary Abs or isotype controls: IgA-FITC (Clone C10-3), B220-PE (Clone RA3-6B2), CD138-APC (clone 281-2), CD5-PE (clone 53-7.3), CXCR5- PE-Cy7 (clone L138D7), PD1-FITC (clone J43), CD11b^−^APC (Clone M1/70), CD86-PE (Clone GL1), CD80-APC (Clone 16-10A1), CD11c-FITC (Clone HL3,), MHC-II-FITC (Clone 39-10-8), CD64-PerCP-Cy5.5 (clone X54-5/7.1), CD103-PE (Clone M290), CD19-PerCP (Clone 6D5), CD45-APC-Cy7 (Clone 30-F11), CD3-FITC (Clone 17A2), CD4-PE-Cy7 (Clone 4-5), TCRβ-PE (Clone H57-597), TCRδ-APC (Clone GL3). For intracellular IL-17A and FoxP3 staining, cells were fixed with Fix/Perm reagents (Cyto Fix/Perm, BD) for 30 min at 4°C and stained with IL-17A-PerCP-Cy5.5 (Clone TC11-18H10), RORγt-PE (Clone B2D) or FoxP3-PE (Clone MF23). Events were acquired on FACSCanto II (BD Biosciences) and were analyzed with Flowjo software version 7.6.1.

### *In vitro* Treg and Th17 Differentiation and Proliferation

Naïve CD4^+^T cells (5 × 10^4^ cells/well) were CFSE labeled and co-cultured with FACS-sorted 24 h-chitosan-DNA-immunized MLN CD11c^+^CD103^+^DCs (1 × 10^4^ cells/well) in medium with 10% FCS in the presence of DNA or chitosan-DNA (5 μg/ml) for 4 days. For Treg differentiation, TGFβ (1 ng/ml) was added. Co-cultured cells were harvested and analyzed for CFSE and intracellular Foxp3 expression by flow cytometry. Cocultured cells were re-stimulated for 6 h with PMA (50 ng/ml, Sigma) and ionomycin (500 ng/ml, Sigma) in the presence of GolgiStop (BD Pharmingen). CD4^+^T cells expressing CFSE and IL-17 were analyzed by flow cytometry.

### RNA Extraction and Real-Time RT-PCR

Frozen MLN, LP, PP, or B cells was homogenized and total RNA was extracted using RNAeasy Kit (Qiagen, Germany). cDNA was prepared by using PrimeScript RT Reagent Kit (Takara, Japan). Relative quantification of indicated genes was determined by real-time PCR with SYBR Green (Takara, Japan). The following primers were used for germline a-transcripts, AID transcripts, TACI, BCMA, BAFFR and GAPDH: α-GT-for, 5′-CCAGGCTAGACAGAGGCAAG-3′; α-GT-Rev, 5′- CGGAAGGGAAGTAATCGTGA-3′; Aicda-for, 5′-AACCCAATTTTCAGATCGCG−3′, Aicda-Rev, 5′-AGCGGTTCCTGGCTATGATAAC−3′; TACI-for, 5′- CCAGGATTGAGGCTAAGTAGCG-3′, TACI-Rev, 5′- GGGGAGTTTGCTTGTGACC−3′; BCMA-for, 5′- CAAGCGTGACCAGTTCAGTGA-3′, BCMA-Rev, 5′-CGATCCGTCAAGCTGACCTG−3′; BAFFR-for, 5′-CACTGGACATACAAGCAGCCT-3′, BAFFR-Rev, 5′- TTCTGAGGAGGGTACAAAGACA−3′; Gapdh-for, 5′- GTGAAGGTCGGTGTGAACGGATT−3′, Gapdh-Rev, 5′- GGAGATGATGACCCTTTTGGCTC−3′. Cycling was performed under the following conditions: denaturation at 95°C for 5 min followed by 45 cycles of PCR (95°C for 10 s, 60°C for 30 s, and 72°C for 10 s). The gene expression was calculated by the software and normalized against GAPDH expression in every reaction. For semi-RT-PCR detection of Aicda, α-GT, Iμ-Cα, gene-specific primers were added, cDNA synthesis and the PCR were performed in the same tube with the following primers: Aicda-For, 5′-ATATGGACAGCCTTCTGATGAAGC-3′, Aicda-Rev, 5′-TCAAAATCCCAACATACGAAATGC-3′; α-GT-For, 5′-CCAGGCATGGTTGAGATAGAGATAG-3′, α-GT-Rev, 5′-GAGCTGGTGGGAGTGTCAGTG-3′; Iμ-Cα-For, 5′-CTCTGGCCTGCTTATTGTTG-3′, Iμ-Cα-Rev, 5′-GAGCTGGTGGGAGTGTCAGTG-3′. α-CT was detected by one-step nested PCR with following primers: Iμ4 (5′-ACCCTGGATGACTTCAGTGT-3′)and Iαup4 (5′-CATCTGGACTCCTCTGCTCA-3′); The next round of PCR was performed using primers as Iα-For (5′-CCAGGCATGGTTGAGATAGAGATAG-3′) and Cμ-Rev (5′-AATGGTGCTGGGCAGGAAGT-3′).

### Statistical Analysis

All values were expressed as means ± SEM. Statistical differences between two groups were determined with the unpaired, two-tailed Student's *t*-test. Data among four groups were analyzed using one-way ANOVA with Bonferroni's *post-hoc* tests. All statistical analyses were performed using GraphPad Prism (Version 5.01). *P* < 0.05 were considered significant.

## Results

### Intranasal Chitosan-DNA Immunization Enhances VP1-Specific IgA Production and IgA CSR of B Cells in the Gut

**Figure 1:**
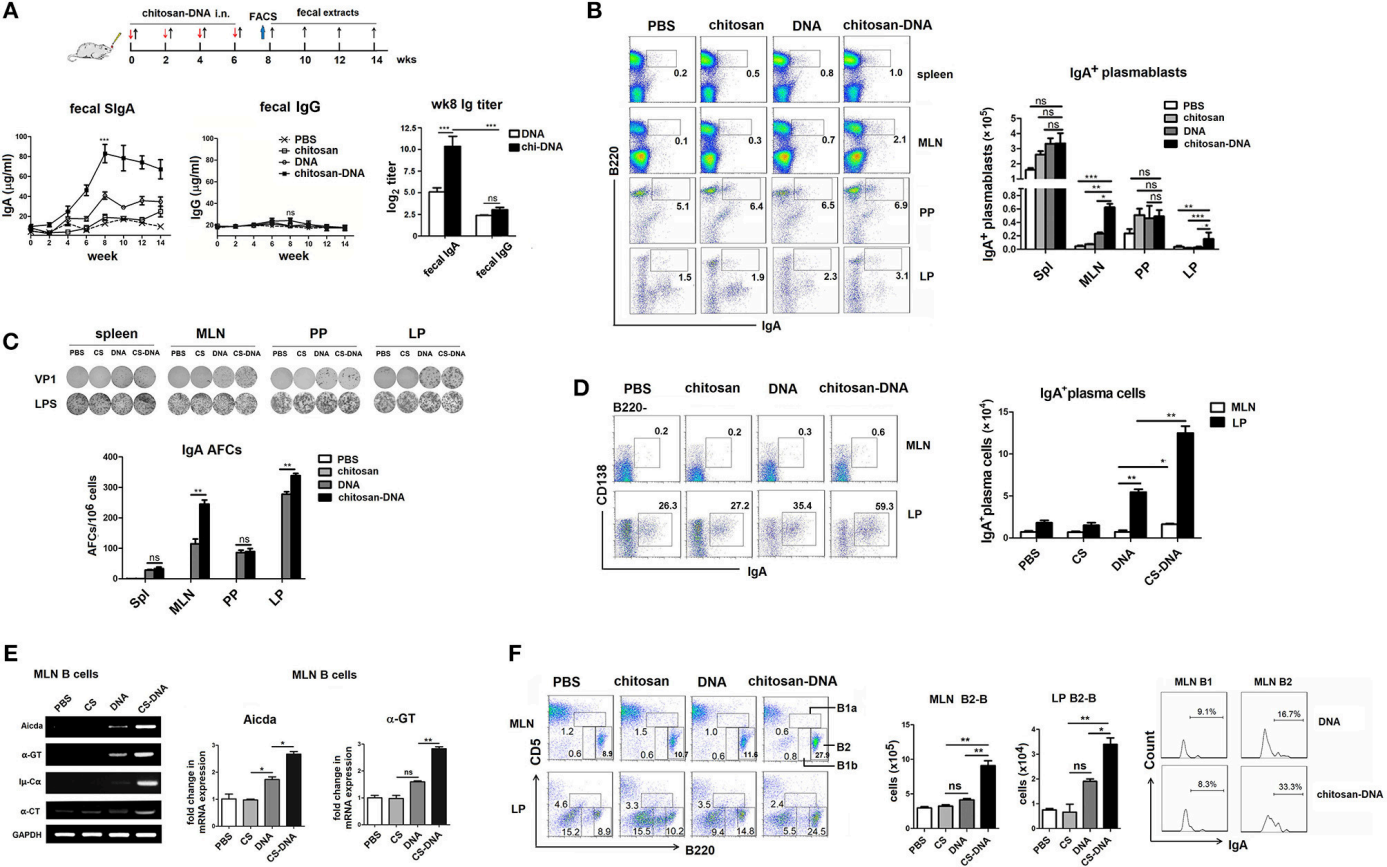
Intranasal immunization with chitosan-formulated DNA enhances specific IgA induction in the gut and B cell IgA CSR in the MLN. **(A)** Mice were immunized intranasally with chitosan, DNA (pcDNA3-VP1, 50 μg) or chitosan-DNA biweekly for 4 times. VP1-specific IgA in fecal extracts was measured by ELISA at indicated time points. **(B)** Representative flow cytometry plots (left) and frequency (right) of B220^+^IgA^+^ plasmablasts from spleens, MLNs, PPs, and LPs of wk8-chitosan-DNA-immunized mice. Data represent mean ± SEM for six individual mice. Data in A and B is representative of three independent experiments. ^***^P < 0.001; ^**^p < 0.01; ^*^P < 0.05; ns, not significant. **(C)** Representative examples of ELISPOT results from wells coated with VP1 peptides for enumeration of VP1-specific VP1-specific IgA antibody-forming cells (AFCs, plasmablasts) in spleen, MLN, PP, and LP of wk8-immunized mice. Experiments were independently repeated three times (*n* = 6). Numbers of AFCs with mean values ±SEM are shown. ^**^p < 0.01; ns, not significant. **(D)** IgA^+^ plasma cells, defined as B220^−^CD138^+^IgA^+^, within the MLN and LP were ensured by flow cytometry, Representative plots were previously gated on B220^−^ cells and frequencies of IgA^+^ plasma cells in MLN and LP with mean values ± SEM were shown (*n* = 6). ^**^p < 0.01; ^*^P < 0.05. **(E)** Representative semi-quantitative (left) or quantitative (right) RT-PCR on sorted B cells from wk8-chitosan-DNA-immunized MLNs for expression of indicated transcripts (Aicda, α-GT, Iμ-Cα, and α-CT). Relative expression of Aicda and α-GT mRNA are displayed as the fold change of respective transcript expression over the expression by Gapdh. Data present mean ± SEM of three independent experiments (*n* = 6). ^**^P < 0.01; ^*^P < 0.05; ns, not significant. **(F)** MLN B cells were subdivided into B1a (B220^lo^CD5^lo^), B1b (B220^lo^CD5^neg^), and B2-B (B220^hi^CD5^neg^) cells according to B220 and CD5 expression. Bar graph represents the absolute number of B2-B cells. Histograms represent expression of mIgA by MLN B1 and B2-B cells after chitosan-DNA immunization. Data present mean ± SEM of three independent experiments (*n* = 6). ^**^P < 0.01; ^*^P < 0.05.

To investigate the effects of chitosan-DNA (pVP1) on gut IgA production, groups of BALB/c mice were intranasally immunized with 4 doses of DNA or chitosan-DNA comprising 50 μg DNA. VP1-specific SIgA and IgG in the fecal extracts were measured by ELISA. VP1-specific SIgA in feces of mice began to increase after two immunizations and reached a peak at wk8. Intranasal chitosan-DNA induced a significantly higher production of specific fecal IgA at wk8 than DNA vaccine did (*P* < 0.001, Figure 1A), whereas there was no difference in fecal IgG level between the two vaccine groups. It indicates that i.n. chitosan-DNA administration enhances VP1-specific IgA production in the intestine.

PPs usually constitute the IgA inductive site while LPs serve as mucosal effector sites. To clarify where chitosan-DNA-enhanced IgA^+^ B response occurs, we examined the IgA-producing B220^+^ plasmablasts in the MLNs, PPs and LPs of mice by FACS, using spleen as a systemic immunity compartment control. Although the highest proportion of plasmablasts was seen in PPs compared to other GALTs of mice, it is not different among various vaccine groups. IgA^+^ plasmablast percentage in MLNs of chitosan-DNA-immunized mice was approximately 3-fold higher than that of DNA-treated mice (Figure 1B). Enumeration of antigen-specific IgA^+^ plasmablasts in various GALTs of mice 2 weeks after final immunization by ELISPOT closely mirrored the results of flow cytometry. VP1-specific Ab-forming cells (AFCs) were weakly induced in the spleens and PPs and were not different among various vaccine groups; however, marked IgA responses (235 and 320 AFCs/10^6^ cells) were observed in the MLNs and LPs of chitosan-DNA-immunized mice that were significantly higher than those induced by DNA (105 and 265 AFCs/10^6^ cells, Figure 1C). Further, IgA^+^ plasma cells, defined as B220^−^CD138^+^IgA^+^ cells, were examined by flow cytometry. Although IgA^+^ plasma cells were predominantly found in LPs but rarely seen in the MLNs of mice; in both places chitosan-DNA induced a higher generation of IgA^+^ plasma cells than DNA vaccine did (*p* < 0.05, Figure 1D). The above data suggest that IgA^+^ plasmablasts, which appear transiently after each vaccination, are predominantly induced in the MLNs, while lately IgA^+^ plasma cells are most efficiently recruited to the LPs of mice by intranasal chitosan-DNA.

Further, the B cell IgA germline transcripts including α-GT, Iμ-Cα, α-circle transcript (α-CT), and expression of AID gene which is a hallmark of ongoing IgA CSR were detected in MLN B cells. MLN B cells from chitosan-DNA-immunized mice showed a significant increase in the expression levels of Aicda, α-GT, Iμ-Cα, and α-CT compared to those from DNA-treated mice as determined by semi-quantitative RT-PCR assay. Consistently, Quantitative RT-PCR of the mRNA level of Aicda and α-GT generated similar results (Figure 1E), confirming the IgA CSR promoting capacity of chitosan-DNA vaccine.

Since mucosal B1 cells are also important source of IgA (35), wk8-immunized MLN B cells were phenotypically devided into B1a (B220^lo^CD5^lo^), B1b (B220^lo^CD5^neg^), and B2 (B220^hi^CD5^neg^) cells, and then monitored their expression of surface IgA (antigen non-specific). B1a or B1b numbers were not different among various vaccine groups (data not shown), whereras MLN B2-B cell numbers were numerically increased by chitosan-DNA immunization, and a 2-fold increase in the proportion of IgA^+^B2 cells was observed in MLNs of chitosan-DNA-treated mice compared with DNA-treated mice (Figure 1F). Taken together, i.n. chitosan-DNA immunization enhances the antigen-specific B2 response undergoing IgA CSR in the MLNs of mice.

### Intranasal Chitosan-DNA Increases Tfh Differentiation Which Correlates With Promoted IgA Titer and Antigen-Specific Plasma Cell Frequency

**Figure 2:**
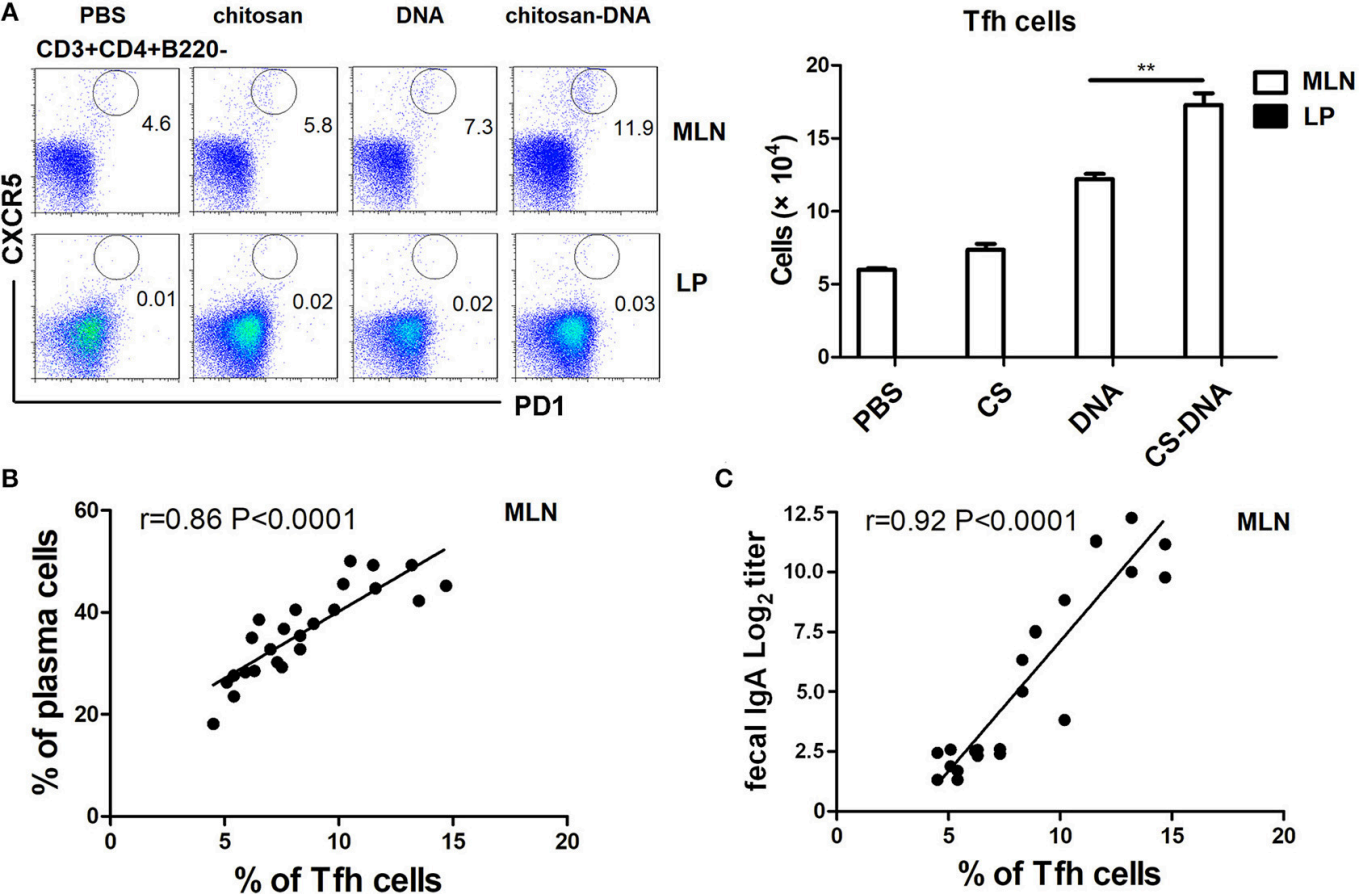
Intranasal chitosan-DNA increased Tfh differentiation in MLN, which correlated with IgA^+^ plasmablast frequency and IgA titer. **(A)** GALT MNC were stained with anti-mouse CD3, CD4, CXCR5, and PD-1 antibodies followed by flow cytometry. Within CD3^+^CD4^+^T cells, Tfh were identified as CXCR5^high^ PD1^high^ T cells. Absolute numbers of Tfh in MLNs and LPs of mice after chitosan-DNA immunization. Data present mean ± SEM of three independent experiments (*n* = 6). ^**^P < 0.01. **(B,C)** Correlation of frequencies of MLN Tfh as determined by flow cytometry and MLN IgA^+^ Plasma cells (% of B cells) (**B**, *r* = 0.86, *n* = 6, *P* < 0.0001) or fecal IgA titer (**C**, *r* = 0.92, *n* = 6, *p* < 0.0001) is shown.

Intestinal Ag-specific IgA is produced as a result of Tfh-B cell interactions in germinal centers (GC). Therefore, we detected the induction of Tfh response by intranasal chitosan-DNA. It was found that GC-resident CXCR5^hi^ PD-1^hi^ Tfh cells, whose function is to promote class switching and somatic hypermutation of naive B cells to produce high affinity IgA, were rarely found in LPs but predominantly present in MLNs. Tfh frequency was significantly increased by chitosan-DNA vaccination as compared with DNA or chitosan treatment (12.0 vs. 7.1 and 5.8% in CD4^+^ cells, Figure 2A). There was a strong correlation between Tfh frequency and IgA^+^plasma cell proportion (Figure 2B) as well as VP1-specific IgA titer (Figure 2C). It indicates that intranasal chitosan-DNA promotes GC Tfh differentiation leading to increased GC B reaction and enhanced IgA CSR.

### Intranasal Chitosan-DNA Immunization Induces an Recruitment of CD103^+^DCs Into the MLNs

**Figure 3:**
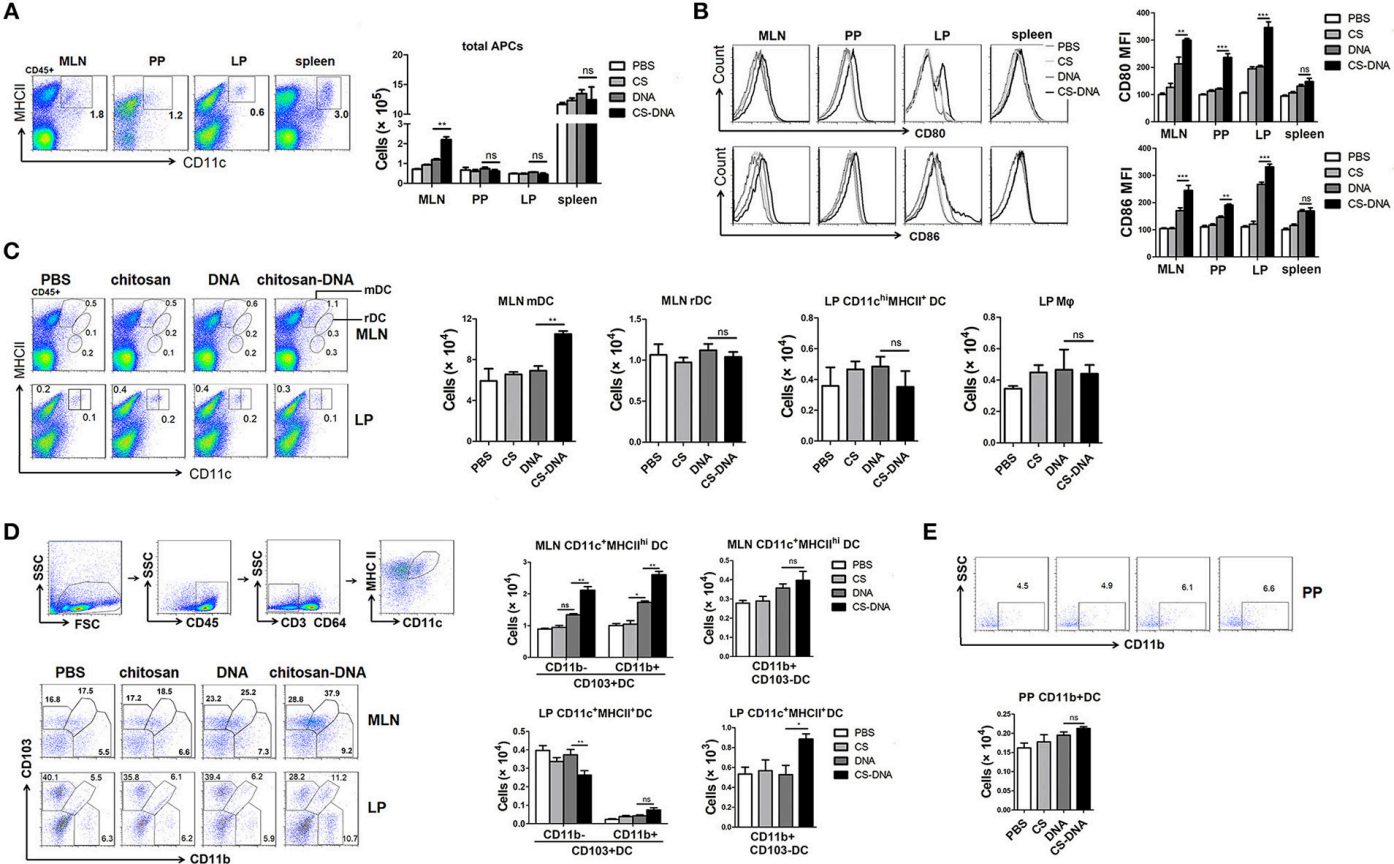
Intranasal chitosan-DNA immunization recruits CD103^+^DCs into the MLNs. **(A)** Representative flow cytometry plots of SI-LP, PP, MLN DC, and spleen DC in BALB/c mice 24 h after DNA or chitosan-DNA immunization. Cells are pre-gated on live, CD45^+^ cells. Absolute cell numbers of CD11c^+^MHCII^+^ cells from various lymphoid organs were summarized in the bar graph. Data are mean ± SEM (*n* = 6) from three independent experiments. ^**^P < 0.01. **(B)** Representative histogram depicting the increase in mean fluorescence intensity of CD80 and CD86 on CD11c^+^MHCII^+^ DCs in various GALTs. Cumulative MFI units data for CD80 and CD86 expression were shown. Data are mean ± SEM (*n* = 6) from three independent experiments. ^***^P < 0.001; ^**^P < 0.01. **(C)** Intestinal DC subset composition. Representative flow cytometry plots (left panel) and cell numbers (right panel) of CD11c^int^MHCII^hi^ MLN migratory DCs (mDCs), CD11c^hi^MHCII^+^ MLN resident DCs (rDCs) and CD11c^hi^MHCII^+^ LP mDCs, CD11c^med^MHCII^+^ LP macrophages from DNA or chitosan-DNA-immunized mice were shown. Data are mean ± SEM (*n* = 6) from three independent experiments. ^**^P < 0.01. **(D)** Representative flow cytometry plots of CD103^+^CD11b^−^DC and CD103^−^CD11b^+^DC subsets after excluding CD64^+^ macrophages from CD11c^+^MHCII^high^ cells. **(E)** CD11c^+^MHCII^+^ PP DCs were further evaluated for expression of CD11b. Cell numbers of CD103^+^CD11b^+^ and CD103^+^CD11b^−^ DC and CD11b^+^DC subsets in the MLNs, LPs and PPs of mice were cumulated in bar graph. Data are mean ± SEM (*n* = 6) from three independent experiments. ^**^P < 0.01; ^*^P < 0.05; ns, not significant.

DCs have critical effects on IgA class switching of B cells (36). To track the DC response after chitosan-DNA immunization, FACS assay was plotted on lymphocytes in the MLNs, PPs, LPs, and spleens of mice. Notably, 24 h after i.n. chitosan-DNA immunization, a significant increase in CD11c^+^MHCII^+^ APCs frequency and numbers (Figure 3A) was only observed in the MLNs, but not in PPs or LPs of mice compared with DNA or chitosan immunization. Chitosan-DNA up-regulated CD80 and CD86 co-stimulatory molecules on DCs from various GALTs (Figure 3B).

MLN DCs can be roughly divided into 3 subsets: MHCII^hi^CD11c^int^ migratory DCs (mDCs), MHCII^int^CD11c^hi^ resident DCs (rDCs) and MHCII^low^CD11c^int^ plasmacytoid DCs (pDCs) (37). Flow cytometry analysis found that intranasal chitosan-DNA had no effect on MLN rDCs numbers, whereas induced a significant increase in proportions and numbers of MLN mDCs (Figure 3C). LP DCs comprise MHCII^+^CD11c^hi^ LP myeloid/migratory DC and MHCII^+^CD11c^int^ LP macrophages (38). No significant differences in SI-LP DC numbers or subsets were observed although chitosan-DNA seemed to decrease proportions of LP-mDCs. 24 h after immunization, after first identifying intestinal mononuclear phagocytes/APCs as CD11c^+^MHCII^+^ cells among live leukocytes and then excluding CD64^+^macrophages, we found three discrete populations based on CD103 and CD11b expression: a majority population of CD103^+^CD11b^+^cDC2 and smaller numbers of CD103^+^CD11b^−^cDC1 and CD103^−^CD11b^+^ DCs. No differences were found in proportion and numbers of CD64^−^CD103^−^CD11b^+^DCs in MLNs. However, a significant increase in frequencies and numbers of CD103^+^CD11b^−^cDC1 and CD103^+^CD11b^+^cDC2 (23.2 to 37.9% within CD64-MHC II^high^CD11c^+^ cells, *p* < 0.01) was induced by i.n. chitosan-DNA as compared to those by DNA vaccine (Figure 3D). In LP, there was a significant decrease in CD103^+^CD11b^−^cDC1 (39.4 to 28.2% within CD64-MHC II^high^CD11c^+^ cells, *p* < 0.01) population, while an increase in CD103^−^CD11b^+^DCs (*p* < 0.05) and CD103^+^CD11b^+^cDC2 (*p* > 0.05) population after chitosan-DNA vaccination. No differences was found in frequencies of CD11b^+^ and CD11b^−^ PP DC subsets (Figure 3E). Taken together, these data indicate that chitosan-DNA antigen sampling induces a migration of intestinal CD103^+^CD11b^−^mDCs from LP to the MLNs of mice 24 h after intranasal immunization.

### MLN CD103^+^DCs Induce IgA CSR in a Th-Dependent Way

**Figure 4:**
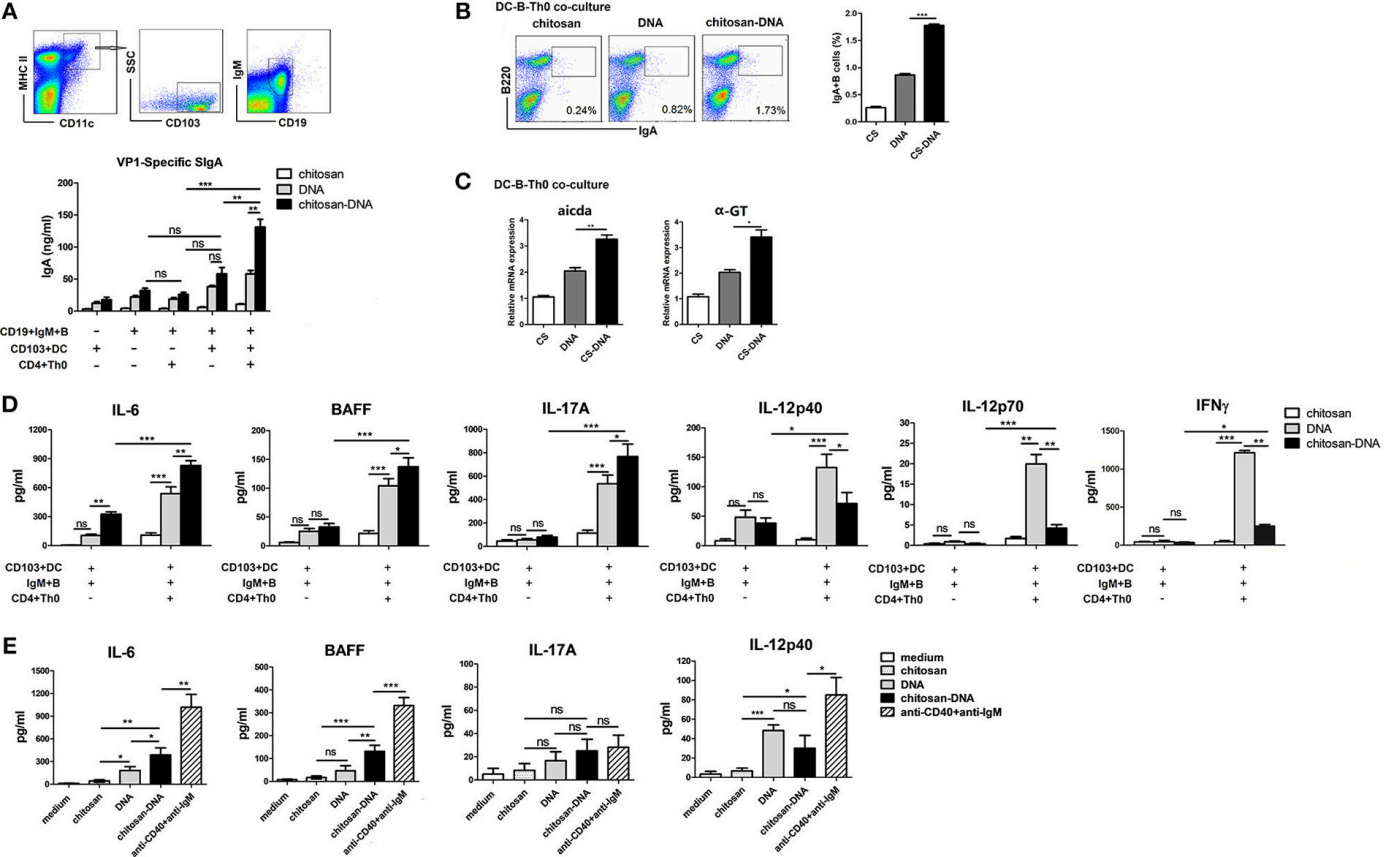
Freshly isolated MLN CD103^+^DCs induce specific IgA production. **(A)** CD103^+^DCs were sorted from 24 h chitosan-DNA-immunized mice. Naïve MLN IgM^+^B cells (5 × 10^5^) were co-cultured with naïve CD4^+^Th0 cells (5 × 10^5^) with FACS-sorted MLN CD103^+^ DCs (2.5 × 10^4^) for 7 days in the presence of DNA or chitosan-DNA (5 ug/ml). VP1-specific IgA secreted in the culture was quantified by ELISA. **(B)** Representative flow cytometry plots of IgA^+^B cells after co-culture and cumulative data quantifying of the number of IgA^+^B cells were shown. Data in **(A,B)** are mean ± SEM representative of three independent experiments. ^***^p < 0.001; ^**^P < 0.01; ns, not significant. **(C)** Quantitative PCR analysis for expression of Aicda and α-GT in co-cultured B cells. mRNA expression is relative to Gapdh. Data are representative of three independent experiments. ^**^P < 0.01; ^*^P < 0.05. **(D,E)** IL-6, BAFF, IL-17A, IL-12p40, IL-12p70, and IFNγ production in the CD103^+^DC-Th0-B co-culture **(D)** or CD103^+^DCs after stimulation **(E)**. MACS-purified naive CD4^+^Th0 cells (5 × 10^5^) were co-cultured with MLN CD103^+^DCs (2.5 × 10^4^) and naïve MLN IgM^+^B cells (5 × 10^5^) for 48 h in the presence of DNA or chitosan-DNA (5 μg/ml). Sorted CD103^+^DCs (1.0 × 10^5^) were incubated with chitosan, DNA, chitosan-DNA (5 μg/ml) or anti-IgM mAb (5 μg/ml) plus anti-CD40 mAb (5 μg/ml). Supernatants were harvested to measure cytokine levels by ELISA at 48 h. Data are representative of three independent experiments. ^***^p < 0.001; ^**^P < 0.01; ^*^p < 0.05; ns, not significant.

We next sought to determine whether CD103^+^DCs were responsible for chitosan-DNA-induced IgA CSR in the intestine. In an *in vitro* culture system, MHC II^+^CD11c^+^CD103^+^cells were flow sorted from MLNs of 24 h-immunized mice, co-cultured with MLN naïve CD19^+^IgM^+^B cells, and naïve CD4^+^Th0 cells at a ratio of 1/20/20 (DC/B/T) in the presence of chitosan-DNA for 7 days. VP1-specific IgA in the culture supernatants was quantified by ELISA. Only background levels of IgA production was observed from B cells if DCs or CD4^+^Th0 cells were absent. Significantly higher production of IgA was observed in the DC-T-B co-culture; and chitosan-DNA-primed CD103^+^DCs induced higher IgA production than DNA-primed CD103^+^DCs (Figure 4A). Flow cytometry analysis of IgA^+^B cells also confirmed higher IgA-secreting B cell frequency in the co-culture with chitosan-DNA-primed CD103^+^DCs than with DNA-primed DCs (Figure 4B). Further, in the DC-B-Th co-culture, increased expression of Aicda and α-GT transcript was detected after chitosan-DNA priming compared with DNA stimulation (Figure 4C).

Then, to see the involved cytokines contributing to the *in vitro* IgA CSR, we investigated the cytokine production by MLN DCs as the APCs co-cultured with naïve CD4^+^Th0 and naïve IgM^+^B cells in the presence of chitosan-DNA. Higher levels of IL-6, BAFF, and IL-17A but reduced levels of IL-12p40, IL-12p70, and IFNγ were produced in co-cultures with chitosan-DNA-primed MLN CD103^+^DCs as APCs compared with DNA-primed DCs (Figure 4D). To see whether the increased IL-6, BAFF, and IL-17A are produced by DCs, we analyzed cytokine secretion from sorted CD103^+^DCs following stimulation with DNA, chitosan-DNA and anti-CD40 mAb plus anti-IgM. It revealed that CD103^+^DCs secreted significant levels of IL-6 and BAFF in response to chitosan-DNA, but low levels of IL-17A (Figure 4E) and IL-23 (data not shown). Since BAFF and APRIL are key cytokines for IgA CSR, we also detected the expression of membrane-bound APRIL (mAPRIL) on MLN CD103^+^DCs 24 h after immunization and found unchanged mAPRIL expression (data not shown). These results indicate that intestinal CD103^+^DCs induce IgA production after sampling chitosan-DNA antigen in a CD4^+^T cell dependent way and that IL-6 and BAFF may be important for the DC-induced IgA secretion.

### MLN CD103^+^DCs Induce IgA Upregulation via IL-6 and TACI Pathways

**Figure 5:**
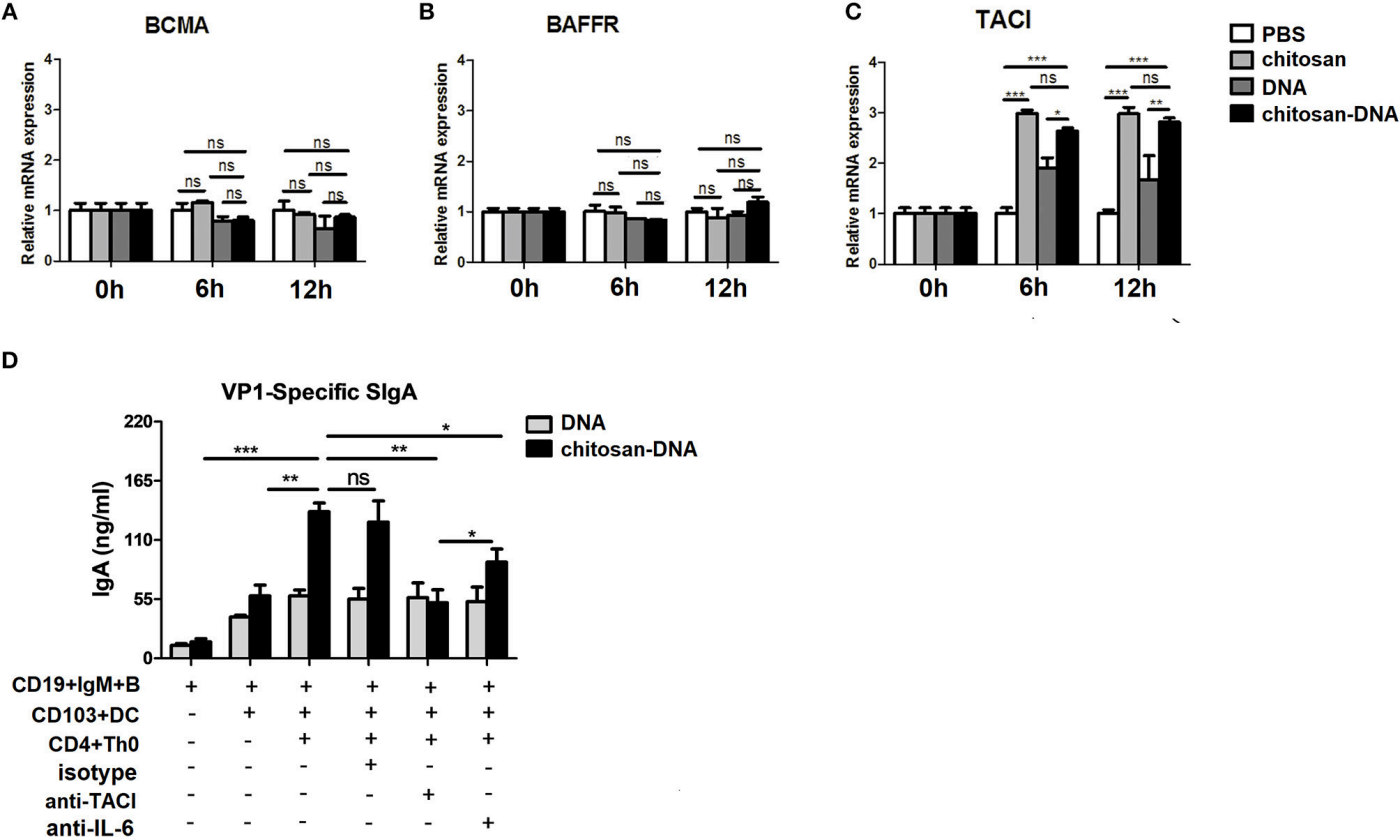
CD103^+^DCs induce specific IgA production in an IL-6 and BAFF-dependent manner. **(A–C)** BAFF receptors expression by immunized MLN B cells. Flow sorted CD19^+^B cells from immunized mice were incubated with chitosan, DNA and chitosan-DNA (5 ug/ml). The mRNA expression of BCMA **(A)**, BAFF-R **(B)** and TACI **(C)** were determined by real-time PCR at indicated time point. Data presented as the mean ± SEM of three independent experiments (*n* = 6). ^***^p < 0.001; ^**^P < 0.01; ^*^P < 0.05; ns, not significant. **(D)** FACS-sorted MLN CD103^+^DCs (2.5 × 10^4^) were primed with DNA or chitosan-DNA (10 ug/ml) and cultured with MLN CD19^+^IgM^+^B cells (5 × 10^5^) and CD4^+^Th0 cells (5 × 10^5^) for 7 days. Either isotype control Ab (10 μg/ml), IL-6-neutralizing Ab (10 μg/ml), or TACI-neutralizing Ab (10 μg/ml) was added to some cultures as indicated. VP1-specific IgA secreted in the culture was quantified by ELISA. Data are mean±SEM from three independent experiments. ^***^P < 0.001; ^**^P < 0.01; ^*^P < 0.05; ns, not significant.

Having established that upon chitosan-DNA immunization, MLN CD103^+^DCs induced IgA CSR in a Th-dependent manner, we further examined the DC mechanisms involved. To clarify the role of BAFF, innate immune mediator that trigger IgA CSR in B cells by engaging the receptor TACI (39), in the induction of IgA, we first examined the expression of BAFFR, BCMA and TACI, candidate BAFF receptor, on sorted B cells from the wk8 chitosan-DNA-immunized mice after incubation with DNA and chitosan-DNA. No significant difference was observed between groups in mRNA levels of BCMA and BAFFR (Figures 5A,B), only TACI level was selectively increased by chitosan and chitosan-DNA incubation (Figure 5C). We then investigated TACI signaling contribution to IgA induction by using anti-TACI Ab. Blockade of TACI totally hampered the chitosan-DNA-enhanced IgA production in B-CD103^+^DC-Th co-culture (Figure 5D). IL-6 has a critical role in inducing IgA within the gut (40). To examine whether IL-6 is responsible for the chitosan-DNA-induced IgA production described in Figure 4A, blocking Ab specific for IL-6 was added to the DC-B-Th cell co-culture system and it partially reduced IgA production in the co-culture (Figure 5D), suggesting IL-6 signaling is involved in the CD103^+^DC-mediated IgA production. Taken together, CD103^+^DCs induce B-cell IgA production via BAFF-TACI and IL-6/IL-6R pathways in response to chitosan-DNA.

### Chitosan-DNA-Primed CD103^+^DCs Promote Th17 Differentiation Which Is Required for the Vaccine-Upregulated IgA Response

**Figure 6:**
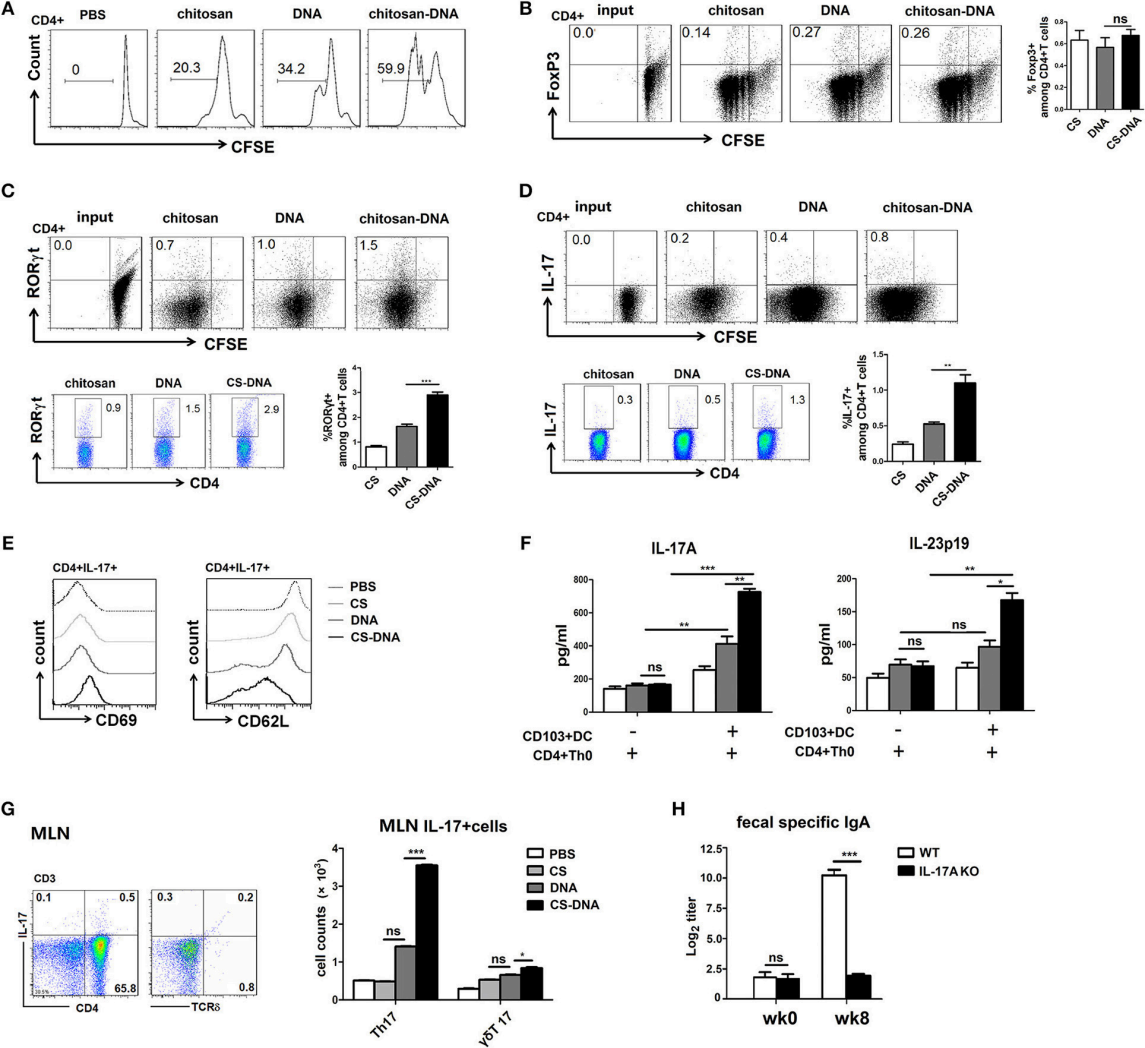
MLN CD103^+^DCs induce T cell proliferation and Th17 differentiation *in vitro* and *in vivo* which are required for IgA induction. **(A)** T cell division after 4-days culture of CD103^+^DCs and CD4^+^Th0. FACS-sorted CD103^+^DCs (1 × 10^4^) from 24-h chitosan-DNA-immunized mice were co-cultured with CFSE-labeled naïve CD4^+^Th0 cells (5 × 10^4^) in the presence of DNA or chitosan-DNA (5 ug/ml), and representative FACS plots of CFSE dilution were shown. **(B–D)** Representative dot plots show Foxp3 **(B)**, RORγt **(C)** and IL-17A **(D)** induction in primed T cells at the start of culture and after 4 d of culture with CD103^+^DCs. T cells were stained for FoxP3, RORγT, IL-17A, and CD4 and analyzed by FACS. The graphs show percentages of Foxp3^+^, RORγt^+^ and IL-17^+^ cells among CD4^+^T cells in the presence of various antigen-pulsed CD103^+^DCs. Data from three independent experiments are summarized as mean ± SEM. ^**^P < 0.01; ^*^P < 0.05; ns, not significant. **(E)** T cell activation was assessed by evaluation expression of CD69 and downregulation of CD62L. Cells were gated as CD3^+^CD4^+^. Representative histograms show the MFI for CD69 and CD62L. Experiments were independently repeated three times (*n* = 3). **(F)** IL-17A and IL-23p19 production in the supernatant were detected by ELISA. Data presented as the mean ± SEM of three independent experiments. ^***^P < 0.001; ^**^P < 0.01; ^*^P < 0.05; ns, not significant. **(G)** MLN lymphocytes were isolated from wk8 chitosan-DNA-immunized mice and gated on CD45^+^CD3^+^ T cells before IL-17A intracellular staining. IL-17A expression by CD4^+^Th and γδT cells was compared among various groups. Representative flow cytometry plots of IL-17^+^cells and cumulative data quantifying of the number of Th17 or γδT17 cells were shown. Data are representative of three independent experiments and presented as the mean ± SEM (*n* = 6). ^***^P < 0.001; ^*^P < 0.05; ns, not significant. **(H)** chitosan-DNA vaccine was i.n. administrated to WT and IL-17A KO mice biweekly for 4 times and the fecal SIgA was detected by ELISA at wk0 and wk8. Data are representative of three independent experiments and presented as the mean ± SEM (*n* = 6). ^***^P < 0.001; ns, not significant.

In addition to releasing cytokines, intestinal CD103^+^DCs moderate gut immune responses in part by inducing regulatory or pro-inflammatory CD4^+^Th cells, which are required for TD IgA CSR (17, 19). In order to address the role of CD103^+^DCs to prime and stimulate naïve CD4^+^T cells *in vitro*, we co-cultured freshly isolated 24 h-chitosan-DNA–immunized MLN CD103^+^DCs with CFSE-labeled naive CD4^+^CD62L^+^T cells in the presence of chitosan-DNA. Following 4-days co-culture, chitosan-pulsed CD103^+^DCs induced similar moderate levels of CFSE dilution as DNA-pulsed DCs, while chitosan-DNA-pulsed CD103^+^DCs were far more efficient at inducing T-cell division (60 vs. 34% and 20% CFSE dilution, Figure 6A).

To investigate the capacity of chitosan-DNA-primed CD103^+^DCs to induce the differentiation of Th17 or Treg cells *in vitro*, flow-sorted 24 h-immunized MLN CD103^+^DCs were co-cultured with CFSE-labeled naïve CD4^+^Th0 cells (1:20 ratio DC/T) for 4 days in the presence of DNA/chitosan-DNA and VP1 peptide, RORγt and Foxp3 expression by CD4^+^T cells were assessed. Compared to DNA priming, chitosan-DNA-pulsed CD103^+^DCs induced the highest frequency of IL-17A^+^RORγt^+^Th17 cells (Figures 6C,D, *p* < 0.001 and *p* < 0.05), whereas no difference in the Foxp3 expression was seen between DNA and chitosan-DNA-pulsed CD103^+^DCs (Figure 6B). Meanwhile, T cell activation was assessed by examining CD69 expression and CD62L downregulation after co-culture (Figure 6E). Compared to PBS and chitosan priming, DNA incubation moderately while chitosan-DNA significantly increased CD69 and down-regulated CD62L expression, indicating that *in vitro* antigen priming by CD103^+^DCs induced T cell activation.

In an attempt to understand why chitosan-DNA-treated CD103^+^DCs were more effective than DNA in priming Th17 cells, we examine the production of Th17-associated cytokines in co-culture supernatant. Chitosan-DNA-pulsed CD103^+^DCs induced higher production of IL-17A and IL-23 by CD4^+^Th cells (Figure 6F), suggesting they drive Th17 differentiation by secreting IL-6 and IL-23 in the MLN. Then we detected IL-17-producing T response in the intestine at wk8 post immunization. Intranasal chitosan-DNA induced elevated total pan IL-17^+^ T response in MLN than DNA vaccine. Specially, Th17 cell numbers in MLNs were significantly higher in chitosan-DNA-treated mice than in DNA-immunized mice (Figure 6G), indicating the preference of chitosan-DNA in inducing Th17 response in the intestine.

As chitosan-DNA favors to induce intestinal Th17 response *in vivo* and *in vitro*, we wish to test whether IL-17 is required for T cell-dependent IgA production achieved by intranasal chitosan-DNA. We i.n. immunized IL-17A–/– and WT control mice with chitosan-DNA and analyzed fecal IgA response. VP1-specific IgA in feces was significantly reduced in IL-17A–/– mice, in contrast to a strong IgA response in WT mice at wk8 post-immunization. (Figure 6H). These results indicate that Th17 cells are required for the IgA switching in MLN after chitosan-DNA immunization.

Taken together, these data show that intranasal chitosan-DNA induces selective specific B cell IgA CSR in MLNs of mice which is dependent upon the recruitment of CD103^+^DCs into MLNs. CD103^+^DCs promotes IgA CSR via increasing the MLN Tfh/Th17 differentiation and via producing BAFF and IL-6. Chitosan-DNA-increased MLN Tfh and Th17 responses are required for the production of antigen-specific IgA.

## Discussion

Here, we demonstrate a key role for intestinal CD103^+^DCs in the induction of T-dependent IgA response in the MLN following intranasal chitosan-DNA vaccination. Chitosan-DNA targets CD103^+^DCs, induces an accumulation of CD103^+^DCs in MLNs 24 h after immunization, and highly induces antigen-specific Tfh and Th17 responses which are indispensable for chitosan-DNA-enhanced IgA CSR. In the MLN, CD103^+^DC-derived IL-6 and BAFF are critical to activate IgA CSR of antigen-specific B cells via IL-6-IL-6R and BAFF-TACI signaling.

Given that most infectious pathogens including highly pathogenic influenza virus, M.tb, and HIV enter the host via mucosal surfaces, there is increasing interest in the establishment of protective mucosal immunity by mucosal vaccination in addition to systemic immunity (24, 41, 42). SIgA production may lead to improved mucosal vaccines (8), and antigen-specific Th17 response is usually associated with enhanced neutralizing SIgA exclusively after mucosal immunization (21). A switched antigen-specific CD4^+^T response from a systemic Th1 to a Th17 dominated mucosa-resident response (43) as well as local IgA response correlate with increased protection against mucosal pathogen infection (44). To solve the necessity to amplify the Th17/IgA responses to a wide array of antigens that are poorly immunogenic at the mucosal sites, cationic polysaccharide chitosan, has been widely utilized as carriers and adjuvants for mucosal vaccines. Meanwhile intranasal vaccination route is adopted since it helps to promote Th17-biased immunity to a variety of different TLR agonists (21, 45). Chitosan-formulated vaccines possess unique advantages like mucosal adhesion, opening the junctions to allow the paracellular transport of antigen, good tolerability, biocompatibility and excellent immune stimulation (46). More importantly, intranasal chitosan-adjuvanted DNA intends to induce an enhanced Th17-biased immune response compared with naked DNA vaccine (Figure 6F) which otherwise strongly increases Th1 immune responses in animals (47). In this regard, intranasal chitosan-DNA nanoparticles represents an effective system for promoting SIgA and Th17 response particularly in the gut, providing enhanced protection against CVB3, an enterovirus (22–24). However, the cellular and molecular mechanism underlying intranasal chitosan-DNA-induced mucosal Th17 response and IgA switching in the gut remains unclear.

PPs are accepted as the principal inductive sites for TD IgA CSR (12, 13). However, MLNs represent alternate IgA inductive site and might be more required for IgA induction in the gastrointestinal tract (20, 48). In our study, to clarify the specific lymphoid tissues targeted by intranasal chitosan-DNA, we compared proportions and counts of migratory DCs (mDC) in various GALTs of mice 24 h after immunization. chitosan-DNA significantly induced an accumulation of CD11c^int^MHCII^hi^ mDCs, and most importantly, CD103^+^ mDCs in the MLN, but not in PP (Figures 3C,D). FACS or ELISPOT analysis of IgA^+^B220^+^ plasmablasts revealed a significant difference in the MLNs and LPs between chitosan-DNA- and DNA-immunized mice (Figures 1B,C). In addition to that, significantly promoted expression of essential hallmarks of active IgA CSR, AID, α-GT, Iμ-Cα and α-CT transcripts were observed in the MLN B cells of chitosan-DNA-treated mice (Figure 1E). Therefore, MLNs are the central target of nasal chitosan-DNA and the inductive sites for vaccine-enhanced IgA CSR.

In the intestinal mucosa, classical DC (cDC) comprise CD103^+^CD11b^−^ (cDC1), CD103^+^CD11b^+^ (cDC2) and CD103^−^CD11b^+^ sub-populations. It has previously been shown that the cCD1 and cDC2 subsets require the transcription factors IRF8 and IRF4, respectively, during their development and differentiation. Each of these subsets plays key, non-redundant roles in controlling immune homeostasis in the intestinal mucosa (49). In commensal and food tolerance, LP-residing CD103^+^DCs migrate to the MLNs where they drive the differentiation of gut-homing FoxP3^+^ Tregs by producing RA (50). Follicular IgA responses also require the migration of antigen-loaded CD103^+^DCs to the interfollicular areas of PPs or MLNs to establish cognate interactions with precursors of Tfh cells, which are essential for the generation of plasma cells. We examined the kinetics of intestinal cDCs after vaccination. Intranasal chitosan-DNA drove a rapid accumulation of both CD103^+^ cDC1 and cDC2 into the MLNs 24 h after immunization. This MLN CD103^+^DC accumulation coincided with a significant decrease in the frequencies and numbers of CD103^+^CD11b^−^ LP-DCs (Figure 3D). Therefore, i.n. chitosan-DNA vaccination induces a migration of cDC1 from the LP to the MLNs of mice and an expansion of cDC2 in the MLNs, both of which may promote Th17/Tfh differentiation and gut SIgA production. Such a LP-to-draining MLNs DC migration could be enhanced by oral, intranasal or intra-peritoneal administration of other adjuvants including TLR agonists (20, 51).

In the draining MLN, gut lumen antigen-sampled CD103^+^DCs play critical roles in the initiation of adaptive Th responses and SIgA production (15, 16, 52). CD103^+^CD11b^+^cDC2 play an important role in inducing Foxp3^+^Treg and Th17 differentiation after sampling oral antigens and migrating to MLN (17, 19). cDC2 are also important in inducing Th2 response (53). Whereas, CD103^+^CD11b^−^cDC1 have been reported to highly generate intestinal IFNγ^+^Th1 cells (54, 55) and also Tregs (56). CD103^+^DC-primed Tregs are associated with antigen-specific IgA induction (20). While recent evidence show that Th17 responses are required for optimal IgA induction (57–59). In consistency with previous report (19), by priming naïve CD4^+^Th0 cells with MLN CD103^+^DCs in the presence of chitosan-DNA and VP1 antigen, we found that chitosan-DNA-primed MLN CD103^+^DCs increased intestinal RORγt^+^IL-17A^+^Th17 cell differentiation and IL-17A/IL-23 production via secreting IL-6 (Figures 6A–E), but decreased DNA-triggered IFNγ production (Th1 response) most probably by reducing DC-secreting IL-12 (Figure 4D). *In vivo*, although we did not detect the change of IFNγ^+^Th1 response, the high production of IL-12 and IFNγ in the supernatant of DC-B-Th co-culture in response to DNA and the nature of DNA vaccine all support that naked DNA strongly induces IFNγ^+^Th1 response *in vivo*. Compared to DNA, intranasal chitosan-DNA enhanced antigen-specific CD4^+^Th17 response in MLN (Figure 6G) while IL-17A deficient mice profoundly impaired fecal IgA response (Figure 6G). Taken together, chitosan-DNA-facilitated Th1-to-Th17 transition via imprinting intestinal CD103^+^DCs plays an essential role in the antigen-specific IgA switching in the intestine.

Intestinal Th17 cells could acquire phenotype of Tfh and induce the development of IgA-producing germinal center B cells (2). The promotion of IgA class switching in GC B cells proves to be a function of ex-Th17 derived Tfh cells, whereas Treg does not adopt a Tfh profile (57). Tfh cells interact with B cells via CD40L-CD40 signaling and via releasing IL-21, IL-4, TGFβ to induce IgA switch (2), thus providing help and critical for the GC B response. Therefore, we detected the induction of Tfh response by chitosan-DNA. A significantly increased CXCR5^hi^ghPD-1^high^ Tfh differentiation was seen in the MLNs by chitosan-DNA immunization than by DNA or chitosan treatment (Figure 2). Tfh frequency correlated with IgA^+^ plasma cell proportion as well as fecal IgA titer, indicating increased GC activity and promoted help from GC Tfh cells achieved by intranasal chitosan-DNA vaccination contribute to increased IgA^+^ B CSR response in the intestine.

Besides presenting luminal antigens to T cells, DCs release cytokines that support local IgA production in a TI manner. TI production of IgA is primarily stimulated by IL-6, RA, TGF-β, BAFF, and APRIL produced by intestinal DC and epithelial cells (60). BAFF and APRIL deliver IgA CSR signals to B cells by engaging TACI, a receptor that signals in cooperation with BCR and TLR ligands (61). TACI promotes the differentiation and survival of plasma cells by binding to MyD88 thereby prompting germline C_H_ genes transcription and AID expression (62). TLR9 and CD40 exhibit a synergistic effect with TACI in promoting Ig CSR (63). BAFF and APRIL cooperate with IL-6 to enhance the survival of IgA-secreting plasmablasts (64). Intestinal CD103^+^DCs or airway epithelial cell-derived IL-6 is considered an important factor to induce IgA response (40, 65, 66). TLR5 and TLR9 ligands (like CpG and DNA) could induce IL-6 production by CD103^+^CD11b^+^DCs (19, 67). While chitosan plus CpG promotes NLRP3-dependent Th17 cytokines production (28). In our study, BAFF (powerful IgA class switch molecule) and IL-6 (an IL-17-inducing cytokine and plasma cell differentiation modulator) were more profoundly secreted by CD103^+^DCs in response to chitosan-DNA than upon DNA directly or after CD4^+^Th0-B-DC co-culture (Figures 4D,E). As a result, IL-17A/IL-23 production in DC-CD4^+^Th0 co-culture was significantly enhanced which also favored IgA switching (Figures 4D, 6F). We further confirmed anti-IL-6 partially, while anti-TACI completely inhibited the IgA secretion in MLN B-Th0-DCs cell co-culture (Figure 5D). Taken together, BAFF-TACI and IL-6-IL-6R signaling considerably contribute to CD103^+^DC-mediated B cell IgA switching upon intranasal chitosan-DNA immunization. Other cytokine like thymic stromal lymphopoietin (TSLP) is thought involved in the activation of myeloid DC and induction of mucosal IgA responses upon mucosal immunization. Nasal CT-adjuvanted PspA vaccine upregulated TSLP and TSLPR expression in the mucosal DCs which promoted IgA-enhancing cytokines (APRIL, BAFF, and IL-6) expression. Whether TSLP-TSLPR signaling is involved in chitosan-adjuvanted DNA-induced IgA promotion needs further study (68).

Taken together, this is the first study to show that intranasal chitosan-DNA promotes IgA CSR of gut B cells via affecting CD103^+^DCs activation and promoting Th17/Tfh differentiation. Chitosan-DNA imprints MLN CD103^+^DCs to stimulate TD IgA switching through the action of two DC/B-cell axes, namely IL-6/IL-6R and BAFF-TACI pathways. Meanwhile, chitosan-DNA decreases CD4^+^Th1 but increases CD4^+^Th17 and Tfh responses in MLN which are required for IgA production. The ability of chitosan-DNA to mobilize intestinal DC migration into the draining lymph node, to promote gut Th17/Tfh responses and to drive SIgA production may explain the exceptional adjuvant properties of this polysaccharide on mucosal immune response. These may together also offer a good basis for the development vaccines with high efficacy against mucosal viruses.

## Author Contributions

WX: conceived and supervised the project, wrote the manuscript. HZ, JY, and MW: performed the experiments. ML and QQ: participated discussion. All authors approved the final version of the paper.

### Conflict of Interest Statement

The authors declare that the research was conducted in the absence of any commercial or financial relationships that could be construed as a potential conflict of interest.

## Abbreviations

αGT: α-germline transcript
AID: Activation-induced cytidine deaminase
APRIL: a proliferation-inducing ligand
BAFF: B cell activating factor of the TNF family
BAFF-R: BAFF receptor
BCMA: B cell maturation antigen
cDC: classical DC
CVB3: Coxsackievirus B3
CSR: Class switch recombination
GALT: Gut-associated lymph tissue
GC: Germinal center
LP: Lamina propria
mDC: migratory DC
MLN: Mesenteric lymph node
PC: Plasma cells
PP: Peyer‘s patch
rDC: resident DC
TACI: Transmembrane activator and CAML interactor
Tfh: T follicular helper
VP1: Viral protein 1.
